# Pertaining to Circumcision

**Published:** 1896-05

**Authors:** I. N. Love

**Affiliations:** Professor Clinical Medicine and Pediatrics, Marion Sims College of Medicine, St. Louis, Mo.; 3642 Lindell Boulevard


### PERTAINING TO CIRCUMCISSION.

BY I. N. LOVE, M. D.

Professor Clinical Medicine and Pediatrics, Marion Sims College of Medicine, St. Louis, Mo.

When I entered the profession, in fact long before I entered it when a youth attending school in St. Louis, growing up as I was, in the family of the leading surgeon of St. Louis, I was impressed by this Sampson in the medical arena, with the importance of circumcision. He took the position that the Mosaic law in this direction was correct; that not only in early life was it conducive to the well-being of the individual, but that all through life it was an advantage. These early impressions have been strengthened with the observation of each succeeding year. This is not the only one of the evidences that we physicians have of the wisdom of the "patriarchs in the house of Israel."

There can be no doubt that the average Jew, while endowed with certainly the average sexual power, and much more than the average disposition to satisfy it, is rarely affected with venereal disease. Jonathan Hutchinson, the great headlight of England, has recently announced that in an experience of half a century, he has never found in a Jew a case of cancer of the penis. Taking the Jew, then, as an example, we certainly have convincing evidence of the value of circumcision as a prophylactic measure.

At the Ninth International Medical Congress, held in Washington in 1887, Dr. Louis A. Sayre favored the dilatation of the prepuce and the breaking up of the adhesions between the same and the glans, and thought that this, in the majority of instances, would be sufficient. At that time I opposed the measure, and favored the more radical one of circumcision, on the ground that if there was an irritation of these parts, a mechanical condition which necessitated interference, the more completely the cause was removed the better; that any measure which was in the nature of palliation or temporizing would result in the directing of the mind of the child toward the part, which should if possible, be kept outside of his philosophy; that the more completely his sexual apparatus was removed from his mental horizon the better. I am stronger than ever in this opinion. The most radical measure, is, in my judgment, the best. I think that a narrowed and constricting prepuce in the boy tends in the direction of stunting the growth of the external organ, which is of vital importance in procreation. By its presence it stimulates the excitability, and just so far does it interfere with the development of his greatest capacity, on the same principle that a hypersensitive nervous system is not a strong nervous system. An organ which has been permitted to grow to its completeness, which is not excited or stimulated in an abnormal way, will certainly have greater capacity for work when called upon.

Mr. Edmund Owen, the fascinating and brainy representative of the surgical diseases of children in England, assures us that the adherence of the prepuce to the glans after birth, is the result of arrested development. He recommends that we draw back the foreskin, lest the lingering adhesions undergo further thickening, as a slight permanent adhesion may cause much discomfort even in cradle life, and later on may suffice to render the affected part irritable and unmanageable. We should impress upon the mother or nurse the thought that a failure to properly appreciate the importance of attention to this organ is a false modesty.

There can be no doubt that a hyper-sensitiveness of the glans penis is an exciting cause of masturbation. Humphrey, Sir James Paget, and that thoroughly practical man, Mr. Lawson Tait, are all strongly favorable to the completest radicalism in the direction of the entire exposure of the glans, and the removal of the redundant tissue which covers it. Tait says that he believes that the practice of masturbation is more of a physical delinquency than a moral evil, and that the best remedy is not to tell the poor children that they are damning their souls, but to tell them that they are seriously injuring their bodies, and to explain to them the nature and purport of the function which they are abusing.

Considering the fact that the operation of circumcision is an insignificant matter in infancy, we should commend it at this time to the parents of children under our care, if there be anything at all tending in the direction of its necessity. I cannot better present my views regarding the operation of circumcision than by quoting Dr. Edmund Owen, who says:

"The patient, under the influence of an anæsthetic, and lying upon a pillow with a thick towel folded beneath the pelvis, should be placed in a good light. The bed is not a convenient place for operating; a dressing table or the top of a low chest of drawers answers well. Having squeezed the blood out of the organ by a gentle compression between the fingers, a small elastic ring, doubled if necessary, and tight enough to control the circulation, is slipped down to the root of the penis. Then, lest the glans be injured, the prepuce is to be drawn forward and held between the blades of the ring dressing forceps and the redundant skin cut off by a large pair of scissors. The mucous membrane, which is closely covering, or perhaps adherent to, the glans, is not implicated in this cut, so that to complete the operation it will be necessary to tear it up along the dorsal aspect by the nails, or by two pair of dissecting forceps. All adhesions between the mucous membrane and the glans must be torn through, and the membrane must be thoroughly reflected, but it is rarely necessary to remove any of it. It should be turned back and stitched to the skin-wound by four or six sutures of fine carbolized gut. If one of these sutures be passed deeply through the frenum the risk of bleeding will be still further diminished, for it is from this fold of membrane that bleeding is most likely to occur. A little strip of dry lint may be lightly wound round the circumference of the end of the penis, and last of all, the india-rubber band is to be divided by a snip of the scissors; this must not be forgotten. The only bleeding that can take place occurs when the band is cut, and as a rule, it merely suffices to cause the dressing to adhere to the wound."

Owen suggests that in order to protect the parts from pressure of the bedclothes a good-sized, firm hat-box, with the bottom knocked out, be made to encircle the pelvis, and by this means the parts are protected from the pressure of the bedclothes. We will find after the operation if there be trouble in passing the water, that it will be accomplished if the little one be permitted to sit in hot water. By the most thorough antiseptic precautions, all that will be necessary will be the simple bathing of the parts in cloths wrung out of cold water.

HEMORRHAGE FOLLOWING CIRCUMCISION.

A hasty summons a short time ago to a youth who had been circumcised in the morning, and left by the surgeon, wherein I found a bed almost completely saturated with blood, a penis wrapped completely about with thoroughly saturated cloths hot with the accumulated heat of the body, the patient almost exsanguinated, impressed me with the importance of the operator seeing before leaving that every possible chance of hemorrhage be removed. A removal of the hot cloths saturated with blood revealed a small vessel bleeding profusely.

A small ligature soon checked it, and instructions were given that the parts be left completely exposed. I would advise after circumcision that the hæmastatic effect of the air be given a full chance, and if after a complete exposure of the parts there be no flow from any particular point within a reasonable time, the operator is safe in leaving. This completely collapsed boy, and the enormous amount of blood with which he was surrounded, impressed me with the thought that even careful surgeons may sometimes overlook the importance of little things.

*3642 Lindell Boulevard.*

